# Bats of the Western Indian Ocean Islands

**DOI:** 10.3390/ani1030259

**Published:** 2011-08-16

**Authors:** John O'Brien

**Affiliations:** Department of Zoology, University College Dublin, Belfield, Dublin 4, Ireland; E-Mail: yosmiley33@gmail.com; Tel.: +353-86-2310-395

**Keywords:** Chiroptera, Western Indian Ocean, fruit bats, ecology, conservation

## Abstract

**Simple Summary:**

The purpose of this paper is to review the literature pertaining to the bat faunas of the western Indian Ocean islands, particularly in light of the identification of many new species on Madagascar and the taxonomic reassignment of others, and to summarise details of their general biology, feeding ecology, reproduction and conservation.

**Abstract:**

The natural colonisation of many remote oceanic islands by bats, including those of the western Indian Ocean, has been facilitated by their unique capability among mammals for powered flight. In the western Indian Ocean region, only the Malagasy islands of Madagascar and the Comoros archipelago have been naturally colonised by non-volant mammals. Despite their greater potential for inter-island dispersal, and thus gene transfer, endemicity of Chiroptera in the western Indian Ocean islands is high. Given their vulnerability to stochastic and anthropogenic disturbances, greater focus needs to be placed on investigating the demographic and ecological history of bats on Western Indian Ocean islands to safeguard not only their future, but also the ecosystem functioning on these islands, for which they are undoubtedly such an integral part. Here, I summarise the taxonomic and life history information available on bats from Western Indian Ocean islands and highlight knowledge gaps and conservation issues that threaten the continued persistence of some species.

## 1. Introduction

The diversity of geological histories for the western Indian Ocean islands, make them a fascinating study of evolutionary history. The granitic Seychelles and Madagascar are continental fragments, as old as the major landmasses themselves. Isolated volcanic mounts, such as Réunion and Mauritius, have a diversity of ages ranging from 2.1 to 15 million years [1]. Coral atolls such as Aldabra and Mayotte, and archipelagos such as the Maldives have undergone repeated inundations due to rising sea levels during the Holocene and so can be considered relatively young. Thus the evolutionary history of bats, and indeed the entire biota, on each island depends on whether the islands in question can be considered isolated ‘oceanic’ islands requiring long-distance aerial or marine dispersal, or have been at some stage attached to larger continental landmasses, facilitating terrestrial dispersal of a vicariant fauna. While phylogeographic studies of this nature are of great interest, there is a pressing need to initiate more in-depth investigations of the general biology of island bats given their conservation concern. Islands are typically characterised by high endemism and reproductive isolation and it is these traits that render island fauna as the most prone to extinction [2].

Here, I consider the Western Indian Ocean as being west of the southern tip of India (not including Sri Lanka) and east of the African coastline (including Madagascar, but not the Mozambique Channel). Typically, the vertebrate fauna of the western Indian Ocean islands is African-derived [3]. However, fluctuations in sea-level, such as during the Pleistocene which exposed much greater land area along the Mid-Oceanic Ridge, certainly afforded easier dispersal opportunities from the Orient [3]. Aerial dispersal from Australasia by means of the trade winds is also theoretically possible [3]. Thus, the extent to which bats have helped shape ecosystem development on these islands depends on the timing of their colonisation, either as ancient or recent dispersers. The large distances between Indian Ocean islands presents an even greater challenge to island colonisation for smaller bats (see below), compared to their fruit bat counterparts. However, Rodrigues and the Maldives are the only islands inhabited by fruit bats that are not currently sympatrically occupied by a smaller representative of the Chiroptera. Bat diversity is greatest on Madagascar and, due to greater research efforts on this island in recent years, new species are being described almost annually. Bats occur on most of the Seychelles archipelago with the exception of some of the outlying islets (e.g., Denis, Bird, Coetivy). Bats have not become established on Chagos, the Amirantes, the Farquhars or Agalega (although occasional vagrants have been recorded), despite bats being present on Aldabra and Cosmoledo.

The 18 families and over 1,000 species of the Order Chiroptera (which is considered to have diversified in the Miocene, about 50 million years ago [4]) have traditionally been divided into two sub-orders–the Megachiroptera (fruit bats or flying foxes) and the Microchiroptera. However, molecular investigation has indicated that the classic grouping of Microchiroptera is, in fact, paraphyletic with the super-family Rhinolophoidea (Rhinolophidae, Hipposideridae, Megadermatidae and Rhinopomatidae) a sister group to the Megachiroptera, forming a Yinpterochiroptera clade, and the remaining species forming the Yangochiroptera clade [4,5], but see [6], and it is this classification system that is followed here.

## 2. Yinpterochiroptera

### 2.1. Distribution, Taxonomy and Putative Origins

Without doubt, *Pteropus* (Pteropodidae) has been the most successful genus in colonising the western Indian Ocean islands (see Appendix Table 1), just as it has been in dispersing throughout the western Pacific from its south-east Asian origins. Typically, a single representative species inhabits each island or archipelago. Only on the islands of Anjouan and Moheli (Comoros archipelago) are two species of *Pteropus* contemporaneously sympatric (*P. livingstonii* and *P. seychellensis comorensis*). However, there is historical evidence that *Pteropus niger* and *Pteropus subniger* (now extinct) were sympatric on both Mauritius and Réunion [7]; sub-fossil evidence that *P. niger* and *Pteropus rodricensis* were sympatric on Mauritius and Rodrigues [7]; and potential sympatry of *Pteropus giganteus ariel* and *Pteropus hypomelanus maris* (a species described based on a single type specimen and now probably extinct) in the southern Maldives. *Pteropus* bats are sympatric with representatives of other large yinpterochiropteran genera including *Eidolon, Rousettus* and *Epomophorus* (all Pteropodidae) on the islands of Pemba, Mafia, Madagascar and the Comoro archipelago. Given, the proximity of the African coast to these islands (in some cases, less than 40 km) it is remarkable that the genus *Pteropus* appears to have never colonised the African mainland.

A molecular study of extant *Pteropus* species [8] provides evidence of at least three colonisation events into the Indian Ocean: an initial colonisation into the westernmost part of the Indian Ocean (Pemba Island and the Comoro archipelago), another giving rise to *P. rodricensis* on Rodrigues, and a final, more recent event, resulting in colonisation of the Seychelles, Aldabra, Madagascar, Mauritius and the Comoro archipelago. Given that Pemba was one of the first islands to be colonised by the genus, this initial colonisation to the westernmost edge of their distribution makes it all the more intriguing that *Pteropus* has not become established on the African mainland itself. A subspecies of *Pteropus seychellensis* (*P. seychellensis comorensis*) occurs on Mafia Island south of Pemba, also within 40 km of the African coast. Monsoon winds that travel south along the African coast could have assisted bats in bridging the gap between Pakistan and India to Pemba and the Comoro archipelago [9]. Dispersal from sub-continental India, utilising the Maldive archipelago as stepping stones, to the Seychelles and beyond is an obvious alternative route of colonisation, albeit difficult to confirm without fossil or sub-fossil evidence. Regrettably, this lack of reliably-dated fossil material for bats from the western Indian Ocean also precludes inferences on the timing of divergence (and thus colonisation) events. However, the last complete inundation of Aldabra atoll is thought to have occurred approximately 125,000 years ago [10], so *Pteropus aldabrensis* and, most likely, its closely-related congeners on Madagascar, Seychelles, Mauritius and the Comoro archipelago can only have diverged after this time. The genetic differences between some species of *Pteropus* in the Western Indian Ocean are exceedingly small, particularly for ‘species’ arising from the most recent colonisation event. In fact, genetic divergence of less than 1.3% for cytochrome *b* gene sequences was reported between some purported species [8]. By way of comparison, mean intra-specific sequence divergence for cytochrome *b* of 1.87% was described between individuals of *Pteropus vampyrus* from two sites in the Philippines [11]. Although genetic evidence suggests that the taxonomic assignment of species for individual islands in the western Indian Ocean may be misleading, the phenotypic differences between island species are suggestive of incipient speciation and so island ‘species’ should be managed separately.

*Rousettus obliviosus* has been recorded from three of the four islands in the Comoro archipelago (Gran Comore, Moheli and Anjouan), while *Rousettus madagascariensis* is endemic to Madagascar where it is widespread except in the south-west of the island. Both of these island endemics, which are sister taxa [12], are smaller in size and have different dentition to the East African mainland species of *Rousettus* (*R. aegyptiacus leachii*) [13], which occurs on Pemba, Unguja (Zanzibar) and Mafia Island [14]. *Rousettus* is considered to have colonised Africa via India or the Middle East through forested corridors [15-17] and arrival on these western Indian Ocean islands is likely to have occurred secondarily to arrival on the African continent. *Epomophorus wahlbergi* is recorded only from Pemba and Unguja among the western Indian Ocean islands, but it occurs throughout eastern Africa. *Epomophorus labiatus (minor)* is recorded from Unguja and Mafia only, but is common and widespread in eastern and central Africa. The genus *Eidolon* is solely African, although this genus does not appear to be closely related to any other African fruit bat [15]. *Eidolon helvum*, a species recorded throughout Africa and into the Near East, is also recorded from Pemba and Unguja, with a congener, *Eidolon dupreanum*, endemic to Madagascar. Sub-fossil remains of *Rousettus* and *Eidolon* from a cave in north-western Madagascar have been dated to approximately 80,000 years ago [18], but certainly this can only be considered a minimum time span for the presence of these genera on this island.

In the western Indian Ocean, rhinolophid bats (Rhinolophidae) only occur on Socotra (*Rhinolophus clivosus)* and the Tanzanian off-shore islands of Pemba, Mafia and Unguja (see Appendix Table 1), where they are clearly associated with the faunal complement of mainland Tanzania (see http://www.fieldmuseum.org/tanzania/Species_Home.asp). *Rhinolophus clivosus* occurs on both the eastern Horn of Africa and the Arabian Peninsula, but it is not clear from which direction it colonised Socotra. Two other species of yinpterochiropteran bats have been described from Socotra Island; *Rhinopoma cystops (hardwickii)* (Rhinopomoatidae) and *Asellia tridens* (Hipposideridae) [19,20]. The genus *Rhinopoma* is thought to have extended its range from the Near East in the Early Miocene and genetic study indicates that colonisation of Socotra occurred from this phylogeographic region [20].

There are seven species in the genus *Triaenops* (Hipposideridae); three that are endemic to Madagascar (*T. auritus, T. furculus* and *T. menamena*); one that occurs only on Aldabra (*T. pauliani*); and three (*T. afer, T. parvus* and *T. persicus)* that occur in an arc from Arabia through to East Africa [21]. Initial genetic evidence supported two independent and unidirectional colonisation events into Madagascar from Africa for *Triaenops* [22], but subsequent analysis indicate one East African colonisation giving rise to *T. auritus* and *T. furculus* and a separate Arabian-derived colonisation for *T. menanmena* [21]. In fact, the morphological/genetic difference between *T. menamena* and the other Indian Ocean forms (*T. auritus, T. furculus* and *T. pauliani*) is so significant that it has been suggested that these latter species be placed in a new genus (*Paratriaenops*) [21]. There is some evidence of geographic partitioning of the sympatric species of *Triaenops* on Madagascar: *T. auritus* is restricted to the north and northwest, *T. furculus* is restricted to the central west and southwest, while *T. menamena* is widespread, but only in the dry habitat of the island [23].

### 2.2. Roosting and General Biology

Most species of *Pteropus* fruit bat favour roosting in emergent trees, which are typically defoliated. Seychelles fruit bats (*P. seychellensis*) roost in tall trees (particularly *Casuarina* or *Albizzia)*, typically hanging from one foot with the other pressed against the ventrum; while *P. aldabrensis* also uses coconut palms, figs and *Sideroxylon inerme* [24]. Although roost sites tend to be in undisturbed forest areas, some species are tolerant of a range of habitat types, such as *P. voeltzkowi* whose roost sites vary from the centre of a village to primary forest and utilises a range of tree species [25], but unlike other species, uses heavily foliated trees. *Eidolon helvum*, the second largest fruit bat on the African continent, typically roosts in emergent trees, but has also been reported to roost in caves [26]. In Kruger National Park in South Africa, *E. wahlbergi* roosts in relatively small groups of mixed sex among foliage in riverine forest [27], while in other parts of their range they favour forest edge habitat or may even be synantrophic (roosts in buildings) [28], but information on roosting behavior on islands appears to be lacking. Generally, roost sites are long-lived; although considerable monthly variation in bat abundance at known *P. rufus* roosting sites (ranges from 10 individuals to rarely 5,000 [29]) in Madagascar has been reported [30]. This ability to move between roosting sites could partially mitigate against anthropogenic threats such as hunting and forest clearance. *Eidolon helvum* is well known for its migratory behaviour and synchrony of roosting behaviour at sites separated by hundreds of kilometres has been described from mainland Africa [31], although whether such synchrony occurs on western Indian Ocean islands is not clear. *Rousettus* bats tend to maintain close body contact when roosting in caves to reduce the energetic costs of homeothermy [13].

*Triaenops furculus* and *T. menamena* primarily roost in caves and frequently co-habit (in fact in an ecomorphological study of variation among Malagasy bats, including four yinpterochiropterans, these two species showed considerable morphological overlap [32]. Large colonies of *T. auritus* have been recorded from a cave and a mining tunnel within its very restricted range [33]. Specimens of *Hipposideros commersoni* are considered to be smaller in the south of Madagascar and this species also shows pronounced sexual selection, with males being larger than females [34]. This species primarily roosts in caves in large colonies, but single specimens have been found to roost in trees and in buildings and are possibly largely inactive during the winter in Madagascar [35]. At least in Egypt, *R. cystops (hardwickii)* typically roost singly in dry caves, underground tunnels and buildings and tend to be active year-round, drawing on fat reserves that begin to appear in late July over the winter [36,37].

Wing clapping is a common behaviour observed among roosting bats and is considered a territorial display and a precursor to antagonistic interactions. Dominance of sympatric *P. livingsonii* over *P. seychellensis comorensis* in aggressive encounters when both species fed from *Ceiba pentandra* trees has been documented [38], but with little other overlap in feeding ecology or in reproductive behaviour between the two species. *Rousettus obliviosus*, which is also sympatric with *P. livingstonii* and *P. seychellensis comorensis* on some of the Comoros islands, does not compete with *Pteropus*, at least for roost sites, since *Pteropus* is tree-roosting, while *Rousettus* roosts in large colonies in caves [39]. In addition, *Rousettus* is smaller and is unlikely to be able to compete aggressively with the much larger *Pteropus* species.

Highly vocal, a range of vocalisations has been described in *P. seychellensis* and olfaction may mediate social contact in all pteropid bats through glands in the neck [24,40]. Male *E. wahlbergi* have air sacs on the neck that may aid in amplifying vocalisations during courtship, and conspicuous scent glands are present in both sexes at the base of the ear and the shoulder epaulets [41]. All species of fruit bat tend to spend large portions of their activity budget in grooming behaviour, particularly focussing on cleaning their wings and removing fruit residues from their fur. Body temperature in fruit bats may be regulated by hanging with the wings outstretched or by licking or urinating on the wings to aid evaporative cooling [42].

### 2.3. Feeding Ecology

*Pteropus* bats utilise fruits as their primary source of nutrition. However, this typically protein poor diet can be supplemented with pollen and leaves to offset these deficiencies. Figs are a consistent food item that arises in dietary analysis of practically all fruit bats in the Western Indian Ocean. Nectar and flowers are also regularly sought and in the process of securing them, bats pollinate trees such as *C. pentandra*, *Pentadesma butyracea* and *Barringtonia sp.* [43]. *P. seychellensis* are commonly observed flying with fruits of *Anacardium occidentale* [24], highlighting their prominent role in seed dispersal. *Guettarda* is an important food source for *P. giganteus ariel* on the Maldive Islands, even though this is quite a low growing plant, bats were observed feeding as low as 1.5m above ground. An unusual food preference in *P. rufus* from Berenty in SE Madagascar has been described where the vast majority of the diet consists of pollen from *Agave sisalana* [44]; a cultivated plant introduced only 60 years ago, thus demonstrating the adaptability and opportunism of feeding behaviour in this genus. In fact, only 16% percentage occurrence of fruit has been reported in the diet of *P. rufus*, compared to 40% pollen and 22% leaf material [44].

Of concern is the preponderance of cultivated food plants in the diet of many fruit bats. An array of cultivated fruits have been described in the diet of *P. giganteus ariel* [43] including starfruit (*Averrhoa carambola*), papaya (*Carica papaya*), banana (*Musa sp.*), guava (*Psidium sp.*), betel nut (*Areca catechu*), among others, with bread fruit (*Atocarpus altilis*) and mango (*Mangifera indica*) causing greatest conflict with fruit growers on the islands. This conflict between fruit growers and fruit bats is repeated on other western Indian Ocean islands, including Mauritius and Madagascar [45]. Despite this reliance on cultivated fruits, in many cases fruit bats remain the most important seed dispersers and pollinators of native plants on these islands.

*Syzygium* is a staple food of *E. helvum* on mainland Africa and they are also said to be particularly fond of *C. pentandra* [46] and given the availability of these foods on the Tanzanian offshore islands, this is likely to be the case there also. This species may also chew on wood and bark [26]. In a study of *E. dupreanum* in eastern Madagascar, 30 plant species (including six introduced species) were recorded in a diet consisting mostly of fruit, but also *Eucalyptus* flowers [47]. Interestingly, this species appeared to ignore plantations of guava (*Psidium sp.*) located close to their roost in favour of native *Polyscias sp.* located further away. Of greater importance, however, is the fact that *E. dupreanum* is most likely the main pollinator of the endangered endemic baobab *Adansonia suarezensis* [48]. Niche partitioning between genera of fruit bats sympatric on islands has not been extensively studied. Although dietary overlap (*Ficus sp., C. pentandra, Musa sp., C. papaya*) has been documented between *Pteropus* and *Rousettus* where they are sympatric, *Rousettus* is predominantly nocturnal in comparison to the more diurnal and crepuscular activity of the *Pteropus* species [39]. In addition, *Rousettus* is a more generalist feeder, foraging in all habitats and altitudinal ranges [39]. In a study of the diets of *Pteropus* and *Eidolon* on Madagascar, of fifty plant species recorded, 23 are consumed in common [49].

Foraging flights for *E. wahlbergi* are generally restricted to a range of about 10 km, despite being a strong flier [50], while some species of *Pteropus* are reported to travel up to 40 km on nightly foraging flights [51]. Typical foraging range for *Eidolon sp.* is up to 30 km from the roost site and often at higher altitudes than most other fruit bats [26]. Gut retention time of seeds could be up to 8 hours in *Pteropus sp.* [52]. Given these long foraging flights and the potential for seed retention, the impact of fruit bats on pollination and seed dispersal over a wide area should not be underestimated.

*Rhinolophus hildebrandti* is reported as venturing up to 2 km from its roost sites and its manoeuvrable wing morphology adapt it to foraging among the canopy [53]. In a study examining five sympatric species (including three yinpterochiropteran bats) from western Madagascar [54], *H. commersoni* was found to feed selectively on Coleoptera (particularly Carabidae and Scarabidae, [55]), while two species of *Triaenops* (*T. menamena* and *T. furculus*) appeared to have a preference for Lepidoptera. However, the authors also indicated that there was some dietary overlap between species and considerable temporal variation in diets. Activity of these five species of bat was greatest at forest edge habitat, coincident with the greatest availability of prey. *Asellia tridens*, a species well suited to desert habitat and recorded only from Socotra among the Indian Ocean islands, are fast and agile fliers, with Lepidoptera and Coleoptera making up a large part of their diet in Israel [56], which is also likely to be the case on Socotra. Echolocation call parameters can suggest some elements of feeding biology; for example, *Cloeotis percivali* echolocates at frequencies exceeding 200 kHz, well beyond the hearing sensitivity of moths on which it predominantly preys [57].

### 2.4. Reproductive Biology

Due to the inherent difficulties in classical observational study of behaviour in bats (large colony size, nocturnal behaviour, and flight capabilities), social structures and reproductive biology are less well-defined as for other mammalian Orders (but see [58] for review). The propensity for multiple mating and post-copulatory delay mechanisms compounds these difficulties. Most *Pteropus* fruit bats can be considered either strongly or moderately gregarious, although the extinct *P. subniger* was reported to inhabit tree hollows singly or in pairs [7]. Harem formation in wild and captive *P. rodricensis* has been reported and there is evidence of resource defence polygyny in captive specimens of this species [59,60], whereby males defend food or roost sites (or both), thereby monopolising reproductive access to females. Spermatogenesis is thought to occur year-round in *Pteropus* bats [61]. Mating has been observed in March, June, October and November in *P. aldabrensis*, but females with young are only reported in December and January. *Pteropus seychellensis* typically copulates in June and July, but mating may be attempted year-round. Births peak in November and December but have also been recorded in September and October and, in one instance, in March [24]. *Pteropus giganteus ariel* has a paturition peak in April and May coinciding with the onset of the rainy season [62], as is also the case with *P. giganteus* on mainland India [63], although young of less than 3 months of age have been observed in November [43]. *Pteropus rufus* is considered to mate in April and May with young appearing in October [36].

Female *Pteropus* fruit bats typically give birth to a single offspring (birth weight for *P. seychellensis* and *P. giganteus* = 31 g [24,64]). Juvenile bats are incapable of foraging until they can fly, and so are nutritionally dependent for a longer period than terrestrial mammals of equivalent size [65]. Thus, despite faster juvenile growth rates compared to other mammals, the rate of population growth is low for mammals of their size due to substantial maternal investment in offspring. Following birth of *P. seychellensis* young in November and December, family groups are the most common types of social groups in roosts, comprising of either a single adult male and female together with dependent young or adult groups of a single male with one or two adult females without young [24]. However, by April, large juvenile amalgamations and single sex groups are more typical, which are maintained throughout the period of the south-east trade winds until females give birth and family groups become re-established.

The timing of reproduction in *R. obliviosus* is unclear, with only incidental observations of roosting young in July, lactating and parous females in July and an embryo in October suggesting polyoestry or some form of pre- or post-copulatory delay mechanism [39]. Mating of *R. aegyptiacus* may occur year-round and births occur, after approximately 4 months gestation, in East Africa in March and September [66], and this is likely to also be the case on the Tanzanian off-shore islands. Mating occurs in *E. helvum* between April and June, and although embryonic development takes four months, gestation periods can be as long as nine months due to delayed implantation [26]. Long distance migration by *E. helvum* into Zambia between October and December is considered to coincide with seasonal fruit abundances and is driven primarily by the energetic demands of pregnancy and lactation [31], and although it is not known if *E. helvum* on the Tanzanian islands migrate, reproductive activity is also likely to be synchronised with food availability. *Epomophorus wahlbergi* has a lek-type mating system with males congregating at ‘arenas’, from where they call to passing females with their shoulder epaulets prominently displayed [41]. Mating can occur twice a year and young are generally recorded around the end of February or from the beginning of October, with gestation lasting 5–6 months [41].

Pregnant females of *R. hildebrandti* have been encountered in September and October, with births occurring in November or December, coinciding with the warm and wet summer months [67,68]. There is evidence of a harem social system in *Hipposideros caffer* (which occurs on Pemba, Unguja and Mafia Island) in colonies studied in Zimbabwe [69]. Reproductive information for *H. commersoni* from Madagascar is lacking.

### 2.5. Conservation

In general, Western Indian Ocean island fruit bats have few natural predators, so predation does not greatly impact on mortality levels in colonies. However, exceptionally high predation on *R. madagascarensis* by barn owls (*Tyto alba*) has been reported at one site in western Madagascar [70].

Fruit bats have been an important traditional food for many island peoples and continue to be utilised, with some species exploited on a commercial basis, such as *P. seychellensis* on the granitic Seychelles and *P. rufus* on Madagascar (360–480 bats taken locally each year, [71]). Unsustainable hunting pressure has been identified as a significant threat to the fruit bats on the Seychelles (J. Gerlach, pers. comm.) and it has been suggested that the IUCN methodology for assigning vulnerability status to bats has significantly underestimated the degree of threat to *P. seychellensis* [72]. Hunting is thought to have been the causative factor in the extinction of *P. niger* on Réunion [73], and until recently, was a major concern on Rodrigues. A detailed study is required of mortality, birth rates, hunting and inter-island movements to determine if a sustainable industry can be established on these islands. Similarly, significant hunting pressure by humans on *H. commersoni* in Madagascar occurs [74], coincident with periods of food shortage. Undoubtedly, incidental capture of other species (including yangochiropterans) also occurs when capturing this species at roost sites.

Conflict with fruit growers is a significant contributory factor to hunting pressures; although the actual scale of damage done by fruit bats has been questioned [71]. *Pteropus rufus* can be legally hunted during a 4-month restricted season in Madagascar, but can also be killed at any time if found raiding crops.

Although it is not yet a concern on Western Indian Ocean islands, the potential for zoonotic disease transmission from fruit bats, may trigger culling programmes in the future. Already, antibodies against the human disease-causing Hendra, Tioma and Nipah viruses have been reported from all three fruit bat species on Madagascar [75].

Currently, the greatest threat to bat populations is habitat alteration. Although some *Pteropus* species appear adaptable to secondary forest and anthropogenic landscapes, concomitant reductions in fruit bat populations generally follow removal of primary forest habitat. The loss of the introduced tamarind tree (*Tamarindus indica*), the fruit of which is a favourite of *P. rodricensis*, has been proposed as a major factor in their decline [7], although this hypothesis has been questioned [76]. Habitat destruction associated with the extension of sugar cane plantations probably contributed to the extinction of *P. subniger* [73]. Thus, protected areas are a vital component of conservation efforts for fruit bats on island habitats, and greater efforts must be made to safeguard current reserves and to establish, extend and enforce new ones where protection is deemed insufficient.

The role that fruit bats play in pollination, seed dispersal and seed set on islands is unequivocal. In a germination study on Madagascar, plant germination was significantly higher from seeds taken from the faeces of either *P. rufus* or *E. dupreanum* in 80% of plants tested [71]. Thus, protecting bat roosting and foraging sites is likely to assist in forest regeneration over even larger areas. On Réunion, all of the native frugivores including two *Pteropus sp.* (*P. niger* and *P. subniger*) have become extinct, except for a bulbul species that can only tackle small fruit. This may be the reason why new lava flows are not being colonised by trees that produce fleshy fruits [73].

Environmental factors can also have a devastating effect on tree-roosting bat populations. Cyclonic storms that strip food and roost trees and sweep bats out to sea have significantly reduced populations of *P. rodricensis* [77]. Deforestation compounds the effects of these storms by reducing the forest buffer that protects bats from the full force of cyclonic winds.

On Pemba, 94% of the *P. voeltzkowi* population is distributed between only 10 roost sites and larger colonies are associated with primary or secondary forest and in one case a graveyard actively protected by locals [25]. Similarly, although *P. giganteus ariel* has been recorded on the majority of islands in the archipelago, group size is generally small (0.6–2.1 bats/hectare) and at least in some cases, visits are likely to be transitory [62]. Both highly localised and low population densities render these bat populations vulnerable to stochastic or environmental factors.

*Cloeotis percivali* appears to be particularly sensitive to disturbance, with large population fluctuations that may be responsible for the stochastic extinctions described from colonies on mainland Africa [78]. The size of the colony of this species on Mafia Island is unknown and warrants further investigation.

Recognising the combined impact that natural perturbations and anthropogenic effects could have on island populations of fruit bats, the IUCN lists one Western Indian Ocean species of *Pteropus* as Critically Endangered *(P. rodricensis)*, two as Endangered *(P. livingstonii, P. niger)* and one Extinct *(P. subniger)* [79]. In October 1989, CITES member states approved the accession of seven species of *Pteropus* bats to Appendix I, and the remaining 53 species were given Appendix II status including all the western Indian Ocean species [80]. Based on their localised distribution and threats from cave disturbance, a revision of the IUCN Red Data List (2002) status of the Comoros endemic *Rousettus obliviosus* from Lower Rick: Near Threatened to Vulnerable was recommended [33], and has since been enacted [79]. *Rousettus madagascariensis* is probably the most numerous species of fruit bat on Madagascar, but still warrants its IUCN listing as Near Threatened due to continued hunting pressure [81].

An IUCN-sanctioned report from 1992 graded all species of Megachiroptera in relation to their conservation status and prioritised the need for conservation action [82]. Three western Indian Ocean *Pteropus* species (*P. livingstonii*, *P. rodricensis* and *P. voeltzkowi*) were given the highest priority rating, indicating that these species are in danger of extinction. Since the report was published, numbers of *P. voeltzkowi* and *P. rodricensis* are growing [76,83,84]. The captive breeding programmes for *P. voeltzkowi* and *P. livingstonii*, recommended in the report, have not had the same success as that for *P. rodricensis*, with only one zoological institution holding an *ex situ* colony of *P. voeltzkowi* (currently only comprising three females) and only four institutions in the *P. livingstonii* breeding programme [85]. The IUCN report further suggested that efforts should be made to introduce *P. rodricensis* to suitable habitat in the Western Indian Ocean islands [82]; a proposal reiterated elsewhere [86]. A similar initiative could be considered for re-introducing *P. niger* onto Réunion in order to restore the large seed-dispersing frugivore fauna now absent from this island, bearing in mind that much of the suitable low-altitude habitat favoured by this species is now gone. However, recent reports suggest that a small group of *P. niger* may, in fact, have already colonised and be breeding on Réunion naturally [87]. The need for national legislative protection, community-based protection and education projects has regularly been highlighted. The former has been enacted in Mauritius and to a lesser extent in Madagascar [71,88], the latter have proven highly successful in the Comoros, Rodrigues and Pemba [89].

Currently, among the non-fruit bat Yinpteropchiroptera inhabiting the Western Indian Ocean islands, only *T. auritus* is listed by the IUCN as Vulnerable due to its restricted range; although populations are considered to be healthy [33]. However, some newly described species have yet to be annotated.

## 3. Yangochiroptera

### 3.1. Distribution, Taxonomy and Putative Origins

The taxonomy of yangochiropteran bats is in a constant state of flux. Thus, the taxonomic names listed in Appendix Table 1 below are currently the most widely accepted, but may be revised in coming years.

Only one species of Yangochiroptera has been recorded from the granitic Seychelle Islands (*Coleura seychellensis*, Emballonuridae). There are only two species described in the genus *Coleura* (Emballonuridae): the widespread *Coleura afra* with a disjointed distribution in low latitudes from southwestern Arabia to western Africa and Madagascar; and the endemic Seychelles sheath-tailed bat (*Coleura seychellensis*). These two species are differentiated morphologically based on a distinctive lip groove in the Seychelles species. Until recently, *C. seychellensis* was probably present on all the larger granitic islands including Praslin and La Digue but can now only be confirmed for Silhouette and Mahé [90]. The genera *Taphozous* (Emballonuridae) and *Chaerephon* (Molossidae) are distributed from SE Asia and Australia westwards to Western Africa. The Mauritian tomb bat (*Taphozous mauritianus*) occurs on Aldabra, Madagascar, Mauritius, Réunion and Unguja and is widespread throughout sub-Saharan Africa, this range correlating with areas of annual rainfall greater than 500 mm [91]. The species complex of *Chaerephon pumilus* (*leucogaster)* is also broadly distributed south of the Sahara desert and on the Indian Ocean islands of Aldabra, Madagascar, Comoros, Pemba and Unguja. Recent and extensive studies of the *Chaerephon pumilus (leucogaster)* species complex have indicated that *C. leucogaster* is nested among lineages of *C. pumilus* [92-94]; although due to the morphological divergence of *C. leucogaster* the authors recommend that it retain its status as a separate species. The species previously referred to as *C. pumilus* occurring in Madagascar is sufficiently differentiated to be considered a separate species (*Chaerephon astinanana*) [94]. *Chaerephon pumilus* sensu lato occurring on the other western Indian Ocean islands (Aldabra [95], the four islands of the Comoro archipelago [96], and possibly the Amirante Islands [97]) are ascribable to *Chaerephon pusillus* [94]. In Madagascar, *C. leucogaster* is restricted to the northern and eastern humid areas up to altitudes of 1,300 m [98], whereas the recently described *C. atsinanana* is common in the eastern areas up to 1,100 m [94]. Likewise, no distinguishing taxonomic differences were found in a comparison of the low-lying savannah species *Mops midas* (Molossidae) from Africa and Madagascar [99]. Indeed, a complete review of the genera *Chaerephon/Tadarida/Mops* is warranted, given the lack of a consistent taxonomy.

Species diversity is greatest on Madagascar, as might be expected, with northern sites of sedimentary rock harbouring most species, although there is no evidence of a north-south cline [100]. Despite having a global distribution, the genus *Eptesicus* (Vespertilionidae) is only represented in the western Indian Ocean by one endemic species in Madagascar (*E. matroka*), although this species has also been assigned to the genus *Neoromicia* (Vespertilionidae) based on bacular morphology [101]. There are two species of the genus *Emballonura* (Emballonuridae) endemic to Madagascar (*E. atarata* and *E. tiavato*) [102]. *Emballonura atarata* is restricted to the moist eastern part of the island, while *E. tiavato* inhabits drier regions. Two other Emballonurid bats also have been documented on Madagascar; *C. afra* and *T. mauritianus* [102]. The Vespertilionidae are well represented on Madagascar with 15 described species (see Appendix Table 1). Four species of *Scotophilus* (Vespertilionidae) have been described from Madagascar (*S. robustus, S. barbonicus, S. leucogaster (viridis)* and the recently described *S. tandrefona*), of which three are endemic [103]. Based on genetic data these *Scotophilus* species are not monophyletic, indicative of multiple colonisation events [104].

Nine species of the genus *Miniopterus* (Miniopteridae) have been described from Madagascar, two of which also occur on the Comoro archipelago, and in some areas of Madagascar, e.g. Namoroka, up to five cryptic species of *Miniopterus* are sympatric [105]. Further taxonomic work may reveal greater cryptic diversity in certain lineages. The enigmatic sucker-footed bats (Family Myzopodidae), endemic to Madagascar and until recently considered monotypic, are represented by two species (*Myzopoda aurita* and *Myzopoda schliemenni*); although *M. aurita* also occurred in East Africa during the Pleistocene [42]. Divergence of these two species has been estimated as occurring approximately 73,500 years ago [106]. *Pipistrellus raceyi* (Vespertilionidae) is known from only four low-elevation locations on Madagascar, two each on the east and west sides of the island [101]. Intriguingly, it has been suggested that only the western populations of *P. raceyi* are forest dependent [107].

On the Mascarene islands (Mauritius, Rodrigues and Réunion), *T. mauritianus, Mormopterus acetabulosus* (Molossidae) and *S. borbonicus (leucogaster)* have all been recorded from Mauritius and Réunion, although *S. borbonicus (leucogaster)* has not been observed on Réunion since 1867, and all are allied to counterparts on Madagascar or the African mainland [73]. *Mormopterus acetabulosus* is thought to be restricted to Madagascar and the Mascarenes, but there are two possible records from South Africa and Ethiopia [108]. There are no recent or historical reports of yangochiropteran bats occurring on Rodrigues or the Maldive archipelago.

*Taphozous mauritianus* has been recorded from the Comoro archipelago; although only as a single specimen from Mayotte [96].

The islands that lie close to the African mainland (Pemba, Unguja and Mafia Island) have subsets of the species complement of nearby Tanzania (see Appendix Table 1), and these islands would benefit greatly from a complete survey focussing on bats. Species diversity appears to be lowest on Mafia Island and highest on Unguja, but with different species assemblages existing on each island [71,109] (see also http://www.fieldmuseum.org/tanzania/Species_Home.asp). An endemic species of the genus *Mops* (*M. bakarii*) has recently been described from Pemba Island [110]. These three islands could, therefore, provide a valuable study on ecological requirements and competitive exclusion for African bats (both yinpterochiropteran and yangochiropteran).

*Hypsugo (Pipistrellus) ariel (bodenheimeri)* (Vespertilionidae) has been recorded from Socotra and thus is the only yangochiropteran representative of the restricted bat fauna here; there is some suggestion that the island population may even be a separate species to that of the mainland [111,112].

### 3.2. Roosting and General Biology

*Coleura seychellensis* typically roosts in groups in caves in boulder fields, either hanging by their hind limbs, or clinging with all four limbs and pressing their abdomen to the rock wall. *Coleura seychellensis* is most active in terms of flight at dawn and dusk and have a preference for coastal and low altitude habitat [113], although a potential high altitude roost site (515 m) in a granite boulder field on Silhouette Island may also exist [114]. In a study of this species on Mahé, it was only encountered on the less well-developed west coast [90]. *Chaerephon leucogaster (pumilus)*, a largely synanthrophic species, roosts in large, stable colonies. Males and females roost together with high site fidelity throughout the year and the social system is suspected to be based on female defence polygyny with some elements of resource defence, at least in western Africa [115]. *Chaerephon leucogaster* and *C. pusillus* are sympatric on the island of Mayotte of the Comoro archipelago and have been captured at the same roost sites [95]. *Taphozous mauritianus* has been recorded from a variety of roost types throughout its range including cliff walls, trunks of large trees, the outer walls of buildings, often in the open but out of direct sunlight [91]. Typically, *T. mauritianus* roosts in small groups of up to 12, with individuals spaced 10–15 cm [116].

Roosting behaviour of yangochiropterans on Madagascar ranges from synantrophy to cave and foliage-roosting and may be seasonal, particularly in the north [117], requiring more long-term studies of bat abundance and movements. *Coleura afra* preferentially roost close to areas of open water, quite often in large colonies, but with distinct and stable clustering of individuals in harems [118]. Both of the *Emballonura* species described from Madagascar are considered forest dependent and typically roost in exposed rock outcrops or caves, although *E. tiavato* is also synantrophic. *Myzopoda aurita* roosts among the unfurled leaves of native *Ravenala madagascariensis* in groups of 9–51 individuals and changes roost site every 1–5 days [119,120]. However, despite a number of specimens of *M. aurita* having been captured, not one female specimen has been obtained and the roosting behaviour of females is unknown [120]. Its sister species, *M. schliemanni*, from the dry western parts of Madagascar has also been described as roosting in *Ravenala madagascariensis* [121], and potentially also in caves [122]. Various cave roosting species may often be found in close association such as *Miniopterus spp.*, *Myotis goudoti*, *Emballonura spp.*, and *Triaenops auritus* [117]; although there is a distinct paucity of investigation into inter-specific interactions between co-roosting species in terms of competition for roost space and territoriality.

An investigation of the habitat use of 10 species of sympatric bats in western Madagascar found that four were predominantly associated with intact humid forests (*Miniopterus manavi*, *Miniopterus majori*, *E. atrata* and *M. goudoti*, the latter species being most strongly associated with this habitat type) [123]. An additional two species favoured agricultural or cultivated land (*E. matroka* and *Neoromica melckorum*) and a further three were synantrophic (*Mormopterus jugularis*, *C. leucogaster* and *Mops lecuostigma*). Taxon richness was highest in the intact humid forest, but intriguingly bat activity was greatest on agricultural or cultivated land, indicating that this habitat type may be a more important foraging resource for all bats. During the austral winter, large numbers of *Mormopterus francoismoutoui* abandon the roost site of Trois Bassins, although it is not known where they move to [108].

Scent-related cues from the inter-aural and muzzle glandular areas mediate sex discrimination and roost-mate recognition for two species of African bat (*Mops condylurus* and *C. leucogaster (pumilus)*, both of which are genera that occur on Madagascar) [124], and individual-specific features of sweep calls by *Otomops martiensseni* allow this species to discriminate between individuals [125], which is probably also the case for *Otomops madagascariensis*. Gular sacs positioned alongside the lower jaw in *T. mauritianus* may function in sexual attraction or stimulation of females [126]. Descriptions of courtship behaviour in yangochiropterans are greatly lacking.

### 3.3. Feeding Biology

All yangochiropteran bats on Western Indian Ocean islands are insectivores. Wing morphology is an excellent predictor of feeding behaviour in bats and available information on wing morphology for some of the yangochiropteran species (or closely-related mainland African forms) from western Indian Ocean islands has been described [127]. For example, *C. leucogaster (pumilus)* is adapted for fast flight in uncluttered (open) terrain and prey is consumed while flying [128], while the more manoeuvrable wing morphology of *Triaenops furculus* is more suited to flying among the forest canopy [129]. Detailed studies of the diets of yangochiropterans from the western Indian Ocean islands have only been carried out for *C. seychellensis* and for some species from Madagascar. Extrapolation of information from closely-related mainland bat species can be misleading since insect assemblages can be quite different on islands compared to the nearest mainland faunal complement. *Coleura seychellensis* forages alone using aerial capture and gleaning over a number of habitat types and, targets a diversity of arthropods, with Coleoptera and Lepidoptera from woodland and Diptera from marshland being the main prey items selected [90,130-132]. Together, these studies suggest some flexibility in foraging strategy, so changes in food availability are unlikely to have been the major contributing factor to population decline in this critically endangered species.

A study of the diets of 3 synantrophic bats (*M. leucostigma, M. jugularis* and *C. leucogaster (pumilus*)) from eastern Madagascar found that Coleoptera, Hemiptera, Lepidoptera and Diptera were the most important insect groups [133]. However, although the proportions of Hemiptera and Lepidoptera in the diet were comparable across all three species, there were differences in Coleoptera and Diptera, with Diptera more frequent in the diet of *M. leucostigma* while Coleoptera were more prevalent in the diet of *M. jugularis* [133]. In a dietary study of sympatric bats in western Madagascar [54], *M. goudoti* favoured Hymenoptera, Neuroptera and Araneae, whereas *M. manavi* focussed on Hemiptera, and both concentrated foraging effort at forest edge habitat.

Male *M. aurita* tend to forage for Lepidoptera and Coleoptera within 1.8 km of their roost site; primarily in coffee plantations, degraded humid forest and wooded grasslands, suggesting this species may be less impacted by habitat change, at least in terms of availability of foraging habitat [119]. However, until the foraging behaviour of females of this species is determined (a female specimen has yet to be captured), the impact of habitat change on this species remains unclear. The obligate cave-dweller *O. madagascariensis* also favours Lepidoptera and Coleoptera, albeit with considerable site variation between the proportions of these two prey items [134].

*Taphozous mauritianus*, generally only encountered flying alone and not higher than the tops of trees at night, is thought to feed primarily on moths found in close proximity to roosts [91]. In a comparative study of foraging behaviour of Molossid bats in South Africa *M. midas*, which also occurs on Madagascar, ventured at least 10 km from its’ roost sites and foraged in the open away from the forest canopy [53] and this is also likely to be the case in Madagascar.

Both *Nycteris thebaica* and *Nycteris grandis* have been reported as generalist feeders, consuming a broad range of invertebrate and vertebrate prey using a variety of hunting strategies [135,136]. In fact, *N. grandis* has been reported as preying on *N. thebaica* on mainland Africa [137], and these two species are also sympatric on Unguja where this predator-prey relationship may also exist.

### 3.4. Reproductive Biology

Apart from investigations into *C. seychellensis* [24,132], the species of yangochiropteran bat in the western Indian Ocean of greatest conservation concern, and on-going research in Madagascar, data on reproductive biology for other island species are largely incidental and anecdotal. The reproductive biology of a range of African bats, including some yangochiroptera occurring on western Indian Ocean islands, has been summarised in a study of the evolution of reproductive patterns and delays [138]. It is likely that reproductive behaviour is not significantly different between island and African mainland forms, where such parapatry occurs.

In general, bats of the Family Molossidae are polyestrous in both tropical and temperate climates, with a decrease in the length of the reproductive season with increasing latitude, although reproductive information on this group from western Indian Ocean islands is greatly lacking. Pregnant females of *O. madagascariensis* have been captured in October in western Madagascar and in November in the south [134].

The non-molossid bats display a tendency towards seasonal or aseasonal polyoestry in tropical latitudes and seasonal monoestry in temperate zones. This division appears to arise more from differences in adaptability to climatic factors in terms of foraging and roosting behaviour as opposed to sexual selection *per se*. Reproductive delays are the exception rather than the norm in tropical latitudes, although reproductive delay at a latitude of 4°S, which is typical for non-molossids only at latitudes greater than 13°N and 15°S, has been described in *C. afra* [138].

Pregnant *C. seychellensis* have been recorded in November and flying young in December and January, suggesting that births occur at the start of the NW monsoon, although mating has also been observed in May suggesting that *C. seychellensis* may be polyoestrus [132]. *Coleura afra* displays post-partum oestrus and highly synchronised parturition after a typical gestation period of 114 days [118,139]. There are two reproductive seasons coinciding with the two rainy seasons in coastal Kenya (March to June and November to December), and although most female *C. afra* give birth during the early rains, a large proportion may not reproduce in the later rainy season [118], and this is probably also the case in the Tanzanian offshore islands. Juveniles born in the later rainy season tend to develop more slowly, but have a higher survival rate than those born in the early rainy season [135]. *Taphozous mauritianus* reproduce throughout the year [138]. *Nycteris grandis* probably give birth in late November and early December [136], at least in the eastern extent of their range, which includes Pemba and Unguja. Mating behaviour in *N. thebaica* involves rapid and erratic flight, together with in-flight head-butting and neck-biting [140]. Implantation occurs in the right uterine horn 16 days after mating and the subsequent gestation period is 2.5–3 months in tropical zones [135].

There is little information on reproductive behaviour for the western Indian Ocean emballonurid bats, but pregnant females of *E. tiavato* have been recorded in mid-December and lactating females in February. *Chaerephon leucogaster (pumilus)* is reported to display year-round polyoestry, producing as many as three litters a year in an 8-month breeding season [115,141], and although the uterus is bicornuate, implantation almost invariably occurs in the right horn [142].

*Myzopoda aurita* appear to segregate sexually, since all colonies described to date only contain male specimens [119]. Thus, the reproductive behaviour of this species from eastern Madagascar remains obscure.

The breeding period of *M. leucostigma* on the islands of Anjouan and Moheli (Comoro archipelago) centers around late November-early December [96].

### 3.5. Conservation

In many cases data is deficient on yangochiropteran species of low density or that are difficult to capture, so estimates of population size may not be accurate, thus making it difficult to assign conservation status and determine management priorities. For example, originally considered rare, *E. atarata* is, in fact, widespread throughout its range [102].

For most yangochiropteran species, disturbance directly at roost sites and/or through habitat alteration is the greatest threat to island bats. For example, the role that human disturbance, particularly in relation to tourist visits to caves, plays in bat abundance and distribution has been documented in Tsingy de Bemaraha National Park in Madagascar [35]. However, the relationship between habitat alteration and conservation status is not straight-forward. As described earlier, cultivated and agricultural lands act as an important habitat type for foraging bats, and many species are synantrophic. Thus, it is important to effect a balance between providing native roosting and foraging habitat for less adaptable species that may be more susceptible to anthropogenic-induced population declines with retaining the mosaic habitat that more opportunistic species may thrive in. In order to achieve this, much greater effort must be invested in determining the ecological parameters that influence bat populations. This also impacts on efforts to delineate conservation areas. Only a few species of yangochiropteran have been described as being forest-dependent (5 of 27 species according to one study [100]) in the dry regions of western Madagascar. Thus, simply protecting the last remaining undisturbed areas of forest in these regions will not be sufficient to adequately buffer non-forest-dependent species from threats elsewhere (e.g., hunting, cave disturbance, *etc.*). However, given that forest edge habitat is clearly a favoured foraging habitat for many bat species [35,54], efforts must continue to safeguard the integrity of forest habitats in Madagascar, not only for its chiropterans, but also its other flora and fauna.

*Coleura seychellensis* is one of only 15 species of yangochiropteran currently listed as Critically Endangered by the IUCN [79]. The total population size of *C. seychellensis* is unlikely to exceed 100 individuals [90,131], with the greatest threats being human disturbance, predation by introduced barn owls and habitat alteration. A reduction in human disturbance, coincident with vegetation management at one roost site, has contributed to an increase in the local population size of *C. seychellensis* [131]. Thus, in order to secure the future of this species and, indeed, to restore self-sustaining populations on the islands it previously inhabited, major habitat restoration projects must be enacted. Such restoration must involve the partial replacement of non-native vegetation, the control of public access to roosting sites and probably more controversially, the control or eradication of non-native predators from at least some of the islands. Investigation of movement between islands would greatly facilitate a workable management plan for this high profile and highly endangered species.

Certain species are of concern because of their restricted distributions e.g., the cave dwelling *Taphozous hildegardeae* is found only on Pemba and Unguja and fewer than ten African coastal localities and is therefore susceptible to disturbance and local extirpation. *Tadarida fulminans* is considered a patchily distributed and uncommon species in Madagascar, with records mostly originating from the central-south region of the island [143].

While conservation efforts for larger Yinpterochiroptera have been effected throughout the western Indian Ocean, the less ‘charismatic’ Yangochiroptera have been largely over-looked. Many of the more recently described species from Madagascar have been recorded within established national parks [111], although this could be an artefact of sampling bias and information is limited on the status of bats outside of these areas. Given that the biology of Yinpterochiroptera and Yangochiroptera are significantly different in many cases, conservation efforts for one group will not necessarily benefit the other. Clearly, greater efforts need to be focussed on protecting known cave roosting sites and synanthropic structures where they are important refuges for these smaller bats. Targeted education programmes, which have been so successful for fruit bats on islands such as Pemba and Rodrigues [89], need to be replicated for the yangochiropteran bats that also play an important role in ecosystem functioning. An update to the 2001 IUCN report ‘*Microchiropteran Bats: Global Status Survey and Conservation Status*’ is needed [144].

## 4. Conclusions

This work provides a synthesis of current data on bats from the islands in the western Indian Ocean. Clearly, not all available information is included, nor are all references cited. However, some obvious concerns are apparent: (1) greater efforts are required to enact legislative safeguards in many island nations to protect both yinpterochiropterand and yangochiropterans from excessive exploitation; (2) research into the fruit bat–fruit grower conflict must be undertaken urgently in terms of the likely economic costs of fruit bat foraging habits and possible mitigation strategies; (3) greater focus on the basic biology of yangochiropteran bats and, in particular, on foraging and reproductive behaviour to better determine conservation requirements; (4) resources must continue to be dedicated to carrying out regular census surveys of bat populations throughout the region to elucidate population cycles and trends; and (5) expand community education programmes throughout the region to encompass all bats.

## Appendix

**Table 1:** Bats of the Western Indian Ocean islands. Seychelles refers to granitic Seychelles (except ^a^). * IUCN Risk data from 2010 IUCN Red List of Threatened Species http://www.iucnredlist.org, Downloaded 05 January 2011. CR = Critical, DD = Data Deficient, EX = Extinct, LC = Least Concern, LRlc = Low Risk least concern, LRnt = Low Risk near threatened, NT = Near Threatened, VU = Vulnerable. ^a^ Reported [145], but no voucher specimens taken. ^a^ Specifically, Picard Island—it has been suggested that references for *T. furculus* on Cosmoledo Atoll were in error [146]. ^b^ Presence questioned [109,110]. ^c^ Restricted to the island of Anjouan. ^d^Known only from a single specimen collected in 1868 at Sarodrano (may well be extinct). ^e^ Although this species is listed as DD by the IUCN, it has not been recorded on Réunion for almost 140 years, despite extensive survey work. ^f^ Reported in western Madagascar [147], but requires verification [101,100]. ^1^Some authors place *T. furculus, T. auritus* and *T. paulani* in a new genus *Paratriaenops*, while the other Malagasy species, *T. menamena*, remains in *Triaenops* [21].

	IUCNRisk*	Madagascar	Seychelles	Aldabra	Mauritius	Rodrigues	Réunion	Comoros	Maldives	Pemba	Unguja	Mafia	Socotra

YINPTEROCHIROPTERA													

**Pteropodidade**													

*Eidolon dupreanum* (Schlegel 1867)	VU	X											
*Eidolon helvum* (Kerr 1792)	NT									X	X	X	

*Epomophorus labiatus (minor)* (Dobson 1880)	LC										X	X	
*Epomophorus wahlbergi* (Sundevall 1846)	LC									X	X	X	

*Pteropus sp.*													
*P. aldabrensis* (True 1893)	VU			X									
*P. giganteus ariel* (Allen 1908)									X				
*P. hypomelanus maris* (Allen 1936)	EX								EX				
*P. livingstonii* (Gray 1866)	EN							X					
*P. niger* (Kerr 1792)	EN				X	EX	X						
*P. rodricensis* (Dobson 1878)	CR				EX	X							
*P. rufus* (Geoffroy 1803)	VU	X											
*P. seychellensis* (Milne-Edwards 1877)	LC		X					X					
*P. subniger* (Kerr 1792)	EX				EX		EX					X	
*P. voeltzkowi* (Matschie 1909)	VU									X			

*Rousettus sp.*													
*R. aegypticus* (Geoffroy 1810)	LC									X	X		
*R. madagascariensis* (Grandidier 1928)	NT	X											
*R. obliviosus* (Kock 1978)	VU							X					

**Rhinolophidae**													

*Rhinolophus sp.*													
*R. clivosus* (Cretzschmar 1828)	LC												
*R. deckenii* (Peters 1867)	NT									X	X		
*R. eloquens* (Andersen 1905)	LC									X	X	X	X
*R. hildebrandti* (Peters 1878)	LC									X			
*R. landeri* (Martin 1838)											X		
*R. swinnyi* (Gough 1908)											X		

**Rhinopomatidae**													

*Rhinopoma cystops (hardwickii) (Thomas 1913)*	LC												X

**Hipposideridae**													

*Asellia tridens (Geoffroy 1813)*	LC												X

*Cloeotis percivali (Thomas 1901)*												X^a^	

*Hipposideros caffer (Sundevall 1846)*										X	X	X	
*Hipposideros commersoni (Geoffroy 1813)*	LC	X											
*Hipposideros vittatus (Peters 1852)*	NT									X			

*Triaenops sp^1^*.													
*T. auritus (Grandidier 1912)*	VU	X											
*T. furculus (Trouessart 1906)*	LC	X											
*T. menamena (rufus)* [148]	LC	X											
*T. pauliani* [146]				X^a^									

YANGOCHIROPTERA													

**Emballonuridae**

*Coleura afra* (Peters 1852)	LC	X								X			
*Coleura seychellensis* (Peters 1868)	CR		X										

*Emballonura atrata* (Peters 1874)	LC	X											
*Emballonura tiavato* [102]	LC	X											

*Taphozous mauritianus* (Geoffroy 1818)	LC	X		X	X		X	X			X		
*Taphozous hildegardeae* (Thomas 1909)	VU									X	X		

**Molossidae**													

*Chaerephon (Tadarida) sp.*													
*C. jobimena* [98]		X											
*C. leucogaster (pumilus)* (Grandidier 1869)	LC	X						X		X	X	X	
*C. pusillus* (Miller 1902)				X				X					
*C. astinanana* [94]		X											

*Mops (Tadarida) sp.*													
*M. bakarii* [110]										X			
*M. brachypterus* (Peters 1852)	LC										X^b^	X	
*M. leucostigma* (Allen 1918)	LC	X						X					
*M. midas* (Sundevall 1843)	LC	X											

*Mormopterus acetabulosus* (Hermann 1804)	VU				X								
*Mormopterus francoismoutoui* [108]							X						
*Mormopterus jugularis* (Peters 1865)	LC	X											

*Otomops madagascariensis* (Dorst 1953)	LC	X											

*Tadarida fulminanas* (Thomas 1903)	LC	X											

**Myzopodidae**													

*Myzopoda aurita* (Milne-Edwards & Grandidier 1878)	LC	X											
*Myzopoda schliemanni* [121]	LC	X											

**Nycteridae**													

*Nycteris sp.*													
*N. grandis* (Peters 1865)	LC									X	X		
*N. hispida* (Schreber 1775)	LC										X		
*N. macrotis (luteola)* (Dobson 1876)	LC										X		
*N. madagascariensis* (Grandidier 1937)	DD	X										X	
*N. thebaica* (Geoffroy 1818)	LC										X		

**Vespertilionidae**													

*Eptesicus (Neoromicia) matroka* (Thomas & Schwann 1905)	LC	X											

*Miniopterus sp.*													
*M. aelleni* [149]		X						X					
*M. brachytragos* [105]		X											
*M. gleni* [150]	LC	X											
*M. griveaudi* (Harrison 1959)	LC	X						X					
*M. mahafaliensis* [105]		X											
*M. majori* (Thomas 1906)	LC	X											
*M. manavi* (Thomas 1906)	DD	X											
*M. minor (Peters 1867)*											X		
*M. petersoni* [151]	LC	X											
*M. sororculus* [152]		X											

*Myotis anjouanensis* (Dorst 1960)	DD							X^c^					
*Myotis goudoti* (Smith 1834)	LC	X											

*Neoromicia sp.*													
*N. (Eptesicus) malagasyensis* [153]	EN	X											
*N. (Pipistrellus) melckorum* (Roberts 1919)	DD	X											

*Pipistrellus sp.*													
*P. grandidieri (capensis)* (Dobson 1876)											X		
*P. hesperidus* (Temminck 1840)	LC	X								X	X		
*P. (Neoromica) nanus* (Peters 1852)	LC	X^f^								X	X	X	
*P. raceyi* [101]	DD	X											
*P. rueppellii* (Fischer 1829)	LC										X		

*Hypsugo (Pipistrellus) anchietae* (Seabra 1900)	LC	X											
*Hypsugo (Pipistrellus) ariel* (Thomas 1904)													X

*Scotophilus sp.*													
*S. cf. borbonicus* (Geoffroy 1803)	DD	X^d^					EX^e^						
*S. marovaza* [103]	LC	X											
*S. robustus* (Milne-Edwards 1881)	LC	X											
*S. tandrefana* [154]	DD	X											
*S. viridis* (Peters 1852)	LC									X		X	
